# Disordered mesoporous silica particles: an emerging platform to deliver proteins to the lungs

**DOI:** 10.1080/10717544.2024.2381340

**Published:** 2024-07-23

**Authors:** Aura Rocío Hernández, Ekaterina Bogdanova, Jesus E. Campos Pacheco, Vitaly Kocherbitov, Mikael Ekström, Georgia Pilkington, Sabrina Valetti

**Affiliations:** a Biofilms – Research Center for Biointerfaces (BRCB), Malmö, Sweden; b Biomedical Science, Faculty of Health and Society, Malmö University, Malmö, Sweden; c Iconovo AB, Lund, Sweden; d Nanologica AB (publ), Södertälje, Sweden

**Keywords:** Dried powder inhalation, protein formulation, micronised drug carrier, mesoporous silica particles, pulmonary drug delivery

## Abstract

Pulmonary delivery and formulation of biologics are among the more complex and growing scientific topics in drug delivery. We herein developed a dry powder formulation using disordered mesoporous silica particles (MSP) as the sole excipient and lysozyme, the most abundant antimicrobial proteins in the airways, as model protein. The MSP had the optimal size for lung deposition (2.43 ± 0.13 µm). A maximum lysozyme loading capacity (0.35 mg/mg) was achieved in 150 mM PBS, which was seven times greater than that in water. After washing and freeze-drying, we obtained a dry powder consisting of spherical, non-aggregated particles, free from residual buffer, or unabsorbed lysozyme. The presence of lysozyme was confirmed by TGA and FT-IR, while N_2_ adsorption/desorption and SAXS analysis indicate that the protein is confined within the internal mesoporous structure. The dry powder exhibited excellent aerodynamic performance (fine particle fraction <5 µm of 70.32%). Lysozyme was released in simulated lung fluid in a sustained kinetics and maintaining high enzymatic activity (71–91%), whereas LYS-MSP were shown to degrade into aggregated nanoparticulate microstructures, reaching almost complete dissolution (93%) within 24 h. MSPs were nontoxic to *in vitro* lung epithelium. The study demonstrates disordered MSP as viable carriers to successfully deliver protein to the lungs, with high deposition and retained activity.

## 1. Introduction

Nowadays, biologics are a significant proportion of therapeutic approaches being explored for the management of diverse diseases owing to their advantageous characteristics such as enhanced safety, target specificity, and pharmacokinetics when compared to conventional small-molecule drugs (Leader et al., 2008). The inhalation route is a noninvasive alternative for delivering proteins and peptides that has been gaining considerable attention because of the possibility to improve patient compliance by replacing invasive injections as traditional biologic treatments (Anselmo et al., 2019) and bypasses potential degradation in the gastrointestinal tract. Pulmonary administration as an entrance for systemic drug delivery has been investigated for decades due to the large, highly vascularized surface area of the lungs and demonstrated permeability to macromolecules (Chung et al., 2012). More recently, attention has been shifted toward the aerosolization of biologics for the local treatment of lung diseases, including respiratory infections, lung cancer and asthma (Liang et al., 2020). However, development of suitable and efficient carrier systems has been a major challenge, especially for reaching the lower airways. In particular, this is due to the complex geometry and physiology of the respiratory tract together with the biological barriers, which interfere with the deposition mechanism, drug instability, and rapid clearance mechanism. Furthermore, the high sensitivity of biologics to various stresses (such as freezing, heat, mechanical shear and concentration-induced osmotic shock) compared to synthetic molecules requires careful consideration of their processing, which can cause degradation and loss in bioactivity. Among inhaled formulations, dry powder formulations are of high interest, largely owing to their user-friendly, portable and inexpensive delivery devices (Chang et al., 2021). However, to ensure required size distribution and protein stability in a dry state, appropriate excipients are necessary, creating a highly challenging landscape.

Particle size is one of the key parameters in the development of dry powder inhalation (DPI) formulations and the respirable fraction is expected to be composed of particles of between 1 and 5 µm (Bosquillon et al., 2001). In this context, several excipients have been studied for improving pharmaceutical formulations and to find the best drug carrier (Liang et al., 2020). The most frequently used carrier in pulmonary drug delivery of small (synthetic) molecules is lactose monohydrate (Karhu et al., 2000). In this approach, commonly known as ordered mixtures, the active ingredient is micronized (1–5 µm) and homogenously mixed with coarse lactose particles (40–100 µm). When aerosolised, the micronised drug is dispersed from the surface of the carrier particles and inhaled, whilst the lactose carriers impacted on the back of the throat and swallowed. However, this approach is generally not suitable for biologics due to instability issue during the process. For instance, there is a general interest to remove lactose because it is a reducing sugar that generate Maillard reaction and therefore could degrade proteins (Karhu et al., 2000; Bosquillon et al., 2001). Another approach is to use a inhalable micronized carrier which encapsulates the active ingredient and delivers it directly to the lungs (Hebbink et al., 2022). The advantage of the latter in the context of biologics is significant. In particular, avoiding the need for micronisation, thus not risking the physical and chemical stability of the encapsulated biologic, is advantageous, as well as ensuring the biologic is protected from other stresses during storage or aerosolisation before its delivery in the lungs. Amorphous, mesoporous silica particles (MSP) are a promising drug carrier for pulmonary targeted release due to their tunable micron particle size, low density (hence facile aerosolisation), high surface area (up to 1300 m^2^/g) and loading capacity (up to 50% w/w of drug content). Furthermore, the mesoporous silica possesses favorable chemical properties enabling functionalization and demonstrated biocompatibility. (For a recent and extensive review in the field refer to (Vallet-Regí et al., 2022)).

In previous drug delivery studies, the most commonly investigated MSP (SBA-15) had generally a pore size around 4 nm diameter, making them unsuitable for the delivery of biomacromolecules, such as DNA or proteins, as the proteins would instead adsorb preferentially on the external surface of the particle and not within the pores. However, more recently, it has been demonstrated that their tunable pore size and facile functionalization of their silanol surface can enhance the adsorption of various biomolecules. In addition, MSP can stabilize encapsulated pharmaceutical drug molecules in their amorphous form, resulting in enhanced solubility and bioavailability (Xia et al., 2012; Valetti et al., 2017). In the case of proteins, MSP have also been demonstrated to improve stability and enhance activity (Kao et al., 2014; Kalantari et al., 2017). Moreover, in the MSP pores, encapsulated biologics are less prone to degradation in body fluids, thus increasing their efficiency (Bruno et al., 2022). Despite this promising profile, the investigation of MSP for inhalation therapy has been hampered by safety concerns in the administration of silica particles to the lungs. It is well known that crystalline silica induces silicosis in the lungs, which is an irreversible, inflammatory pulmonary disease. In contrast in vivo subchronic inhalation toxicity studies have shown that while crystalline silica induces persistent granulomas, aerosols of synthetic amorphous silica do not cause fibrosis in the lung (Reuzel et al., 1991; Weber et al., 2018). Moreover, adverse effects after inhalation of amorphous silica have been shown to be partly or completely regressed after recovery. This is likely due to the degradability of amorphous silica in the lung fluid (Braun et al., 2016).

Additionally, another interesting feature of MSP is that depending on their pore structure, they can be classified as ordered and disordered. Ordered MSP have a specific pore structure with narrow size distribution, whilst the disordered MSP typically have a broader pore size distribution, without a specific shape, consisting of an interconnected microporosity, with hierarchically non-ordered pores. Thanks to the larger pore size, disordered MSP have shown some advantages allowing higher drug loadings and faster release for poorly soluble compounds, such as indomethacin (Limnell et al., 2011) and clofazimine (Valetti et al., 2017; Angiolini et al., 2018). Furthermore, disordered MSP offer a totally amorphous material in contrast to hierarchically ordered MSP (Tahir et al., 2018), important property for a safety prospective in lung drug delivery, as explained above. Consequently, exploring the use of disordered MSP as carriers for proteins to the lungs is a valuable and promising approach for localized inhalation therapies.

Motivated by these findings, the aim of this work was to investigate the use of disordered MSP as micron-sized carriers to obtain a free-flowing dried powder inhalation (DPI) formulations for the delivery of proteins. As model protein, lysozyme (LYS), which is a polypeptide with a molecular weight of 14.3 kDa and globular structure. This specific protein was chosen since it is a well-studied in protein with known structure and physical-chemical properties and one of the most abundant antimicrobial proteins in the human airways (Dajani et al., 2005).

The structure of this study is as follows. In the first part, we optimize the conditions for lysozyme loading into MSP (i.e. LYS-MSP), in particular the influence of the ionic strength of the buffer media used for lysozyme adsorption. Next, we investigate the pore filling of the obtained LYS-MSP formulations by N_2_ sorption/desorption and small angle X-ray scattering, as well as evaluate the morphology of the particles in SEM and their thermal properties. Based on these solid-state characterizations, one formulation was selected for the aerodynamic particle size distribution characterization using the Next Generation Impactor (NGI). Subsequently, we studied the release profile and the enzymatic activity of lysozyme released from the LSP-MSP in simulated lung fluid. Finally, we examined the toxicity of the MSP carrier on lung epithelial cell lines.

## 2. Materials and methods

### 2.1. Materials

Lyophilized lysozyme (LYS) from chicken egg white (CAS 12650-88-3, protein ≥90%, ≥40,000 units/mg protein) and Phosphate Buffer Saline (PBS) tablets were purchased from Sigma-Aldrich Sweden AB (Stockholm, Sweden). Simulated lung fluid (SLF), or Gamble’s solution, was prepared as described in Marques et al. (2011). Milli-Q (MQ, ELGA, Purelab Flex, 18.2 MΩ.cm) water was used for all preparations. The mesoporous silica particles (MSP) used in this study were synthesized by Nanologica AB (publ) using their proprietary sol-gel method (Chunfang Zhou et al., 2018).

### 2.2. Adsorption of lysozyme on MSPs

Serial concentrations 0.01–5.00 mg/mL of LYS were prepared in PBS pH 7.4 at three different ionic strengths 15, 50 and 150 mM. 10 mg of MSP were mixed with 5 g of LYS solutions and the resulting mixtures were kept under orbital shaking (240 rpm) for 24 h at 25 °C an Aqua Pro Shaking Water Bath (OLS). Subsequently, the samples were centrifuged at 1500 G for 10 min and the pellets transferred in glass round bottom flasks, placed at −80 °C for 24 h and then freeze-dried using Alpha 1–4 LSC, CHRIST. The supernatants were collected to quantify the unabsorbed LYS by UV-vis analysis at 281 nm wavelength (UV 1800 spectrophotometer Shimadzu, Japan). A calibration curve was obtained from dilution series in PBS (pH 7.4) with nine concentrations between 0.01 and 5 mg/mL. The criteria for qualifying calibration levels was calibration verification (CV) less than 10% for being accepted. The level of quantification (LOQ) was 15.81 µg/mL. The adsorbed LYS was quantified indirectly from the mass balance, as well as from TGA and HPLC (see Sections 2.4.3 and 2.7).

### 2.3. Preparation of the lysozyme dried powder formulation (LYS-MSP) for inhalation

MSP (2 mg/mL) were added to LYS solution (2.5 mg/mL) in 50 mM, 150 mM PBS buffer and were kept under shaking (100 rpm) for at least 5 h to allow for adsorption to occur. The supernatant was then removed by centrifugation and the pellet freeze-dried (see details in Section 2.2), obtaining the sample named ‘LYS-MSP’. To remove any residual of buffer, and potentially non-encapsulated lysozyme, the pellet was washed with 2 mL of Milli-Q water, centrifuged, resuspended in 2 mL of fresh Milli-Q water and freeze-dried, obtaining the sample named ‘LYS-MSP-w’. Finally, two samples, labeled as ‘MSP placebo’ and ‘MSP placebo-w’ were prepared in the same way as the ‘LYS-MSP’, but without lysozyme, without and with a subsequent washing step, respectively.

### 2.4. Solid-state analysis of the lysozyme formulation

#### 2.4.1. Pore size distribution analysis

Nitrogen adsorption/desorption analysis was performed using a TriStar II 3020 (Micromeritics Instrument Corporation, Norcross, GA, USA). The pre-drying and degassing of the unloaded MSP was performed at 200 °C for 6 h. For all LYS-MSP formulations, a lower temperature pre-drying approach was conducted at 40 °C for 24 h to avoid LYS denaturation. The specific surface area was determined through the BET model (Brunauer et al., 1938) and the pore size dimensions were calculated using the Barret-Joyner-Halenda (BJH) model (Barrett et al., 1951), using the desorption curves. Total pore volume (V_pore_) was determined at a relative pressure (P/P_0_) > 0.98.

#### 2.4.2. SAXS

Small-angle X-ray scattering (SAXS) experiments were carried out at the CoSAXS station at the MAX IV synchrotron (Lund, Sweden). All measurements were conducted using glass capillaries (diameter 1.5 mm). The SAXS experiments were performed using an X-ray energy of 12.4 keV, corresponding to a wavelength of 0.1 nm. Two-dimensional SAXS images were recorded using the EIGER2 X 4 M detector (Dectris AG, Switzerland) located at a sample-to-detector distance of 2.31 m. The experimental *q*-range was 0.05–10 nm^−1^.

The scattering from an empty capillary was measured using the same setup and subsequently subtracted as a background from the sample scattering curves. The scattering curves were fitted in SaSView using the correlation length model. This model describes scattering signal as composed of two parts (Hammouda et al., 2004): Porod power-law and scattering from polymer chains. Together, using these two components, the scattering intensity is expressed as:

(1) $$I\;(q)=\frac{A}{q^{n}}+\frac{C}{1+{\left(q\xi \right)}^{m}}+\text{background}$$

where I is scattering intensity, q is scattering vector, A is Porod scalling factor, C is Lorentzian Scaling Factor, ξ is a correlation length for the polymer chains. In-house SAXS experiments were also conducted on an XEUSS 3.0 with coper source (1.54 Å). Two-dimensional SAXS images were recorded using a PILATUS 300K detector, located at a distance of 70 and 400 mm to the sample. The scattering vector $q=2\pi \text{sin}\;\frac{2\theta }{\lambda }$, where λ is the wavelength and 2θ is the scattering angle, was calibrated with a silver behenate sample. Reported scattering profiles *I*(*q*) were obtained by radially averaging the 2D SAXS images. The experimental *q*-range was 0.1– 5 nm^−1^.

#### 2.4.3. Thermogravimetric analysis (TGA)

TGA (TA Q500, New Castle DE, USA) was performed to quantify the amount of water, salts and lysozyme in the dried formulations. Samples (5–10 mg) were loaded into an open platinum pan and heated from 25 °C to 1000 °C at a temperature ramp of 10 °C/min, 3 repeats were performed per sample. Lysozyme undergoes chemical decomposition in the temperature range (200–800 °C). Also, within the same temperature range, silanol groups of MSP undergo condensation reaction with water formation (Lochmuller & Kersey, 1988). We assume that the impact of water formation from the presence of these silanol groups (2.2 wt %) is constant for all formulations and the weight loss for an equivalent mass of unloaded MSP over the same temperature range (200C800 °C) was subtracted for all samples.

#### 2.4.4. Scanning electron microscopy (SEM)

SEM imaging was conducted with an EVO LS10 (Zeiss, Oberkochen, Germany) scanning electron microscope (SEM). The samples were mounted on an SEM stub with double-sided Leit adhesive carbon tape (Agar Scientific) and coated with gold to a nanosized thickness using a sputter coater (Agar Scientific, Essex, UK) at 30 mA and 0.08 mbar pressure for a total sputtering time of 40 s. Imaging was conducted in high vacuum mode, using a secondary electron detector, at 15 kV accelerating voltage and 50 pA probe current.

#### 2.4.5. ATR-FTIR

The pure lysozyme, empty MSPs, LYS-MSPs were analyzed with a Thermo Nicolet 6400 spectrometer (Thermo Fisher, Waltham, USA) equipped with a liquid-nitrogen-cooled MCT-A detector (Thermo Fisher, Waltham, USA). A Smart iTR accessory (Thermo Fisher, Waltham, USA) was used to acquire ATR-FTIR spectra. Each ATR-FTIR spectrum was obtained by conducting 100 scans at a resolution of 8 cm^−1^ in the wavenumber range of 4000–400 cm^−1^. To calibrate for ambient air and absorbance of the diamond, a new background was acquired prior to each new spectrum collection.

### 2.5. Carrier degradability, protein release and activity in simulated lung fluid

Freeze dried samples LYS-MSP loaded in 50 mM PBS and subsequently washed (LYS-MSP-w, 10 mg) were suspended in SLF (5 mL) at 25 °C under orbital shaking at 240 rpm an Aqua Pro Shaking Water Bath (OLS). To study the release kinetics, an aliquot of 2 mL was withdrawn and immediately replaced with fresh medium, at each time point until 72 h. The lysozyme released was measured by spectrophotometry technique, as previously mentioned in Section 2.2.

The weight fraction of released lysozyme ($\omega_{\text{released},i}$) at each specific interval was calculated as follows:

(2) $$\omega_{\text{released},i}=\frac{m_{\text{released},i}}{m_{\text{loaded}}}=\frac{C_{i}\cdot m_{i}}{\omega_{\text{lys}}\cdot m_{\text{sample}}}$$

where $m_{\text{released},i}$ is the mass of released lysozyme, $C_{i}$ is the concentration of released lysozyme determined by UV-spectrophotometry, $m_{i}$ is the total mass of solution, $\omega_{\text{lys}}$ is the weight fraction of lysozyme in the dried powder formulation, $m_{\text{sample}}$ is the mass of dried powder formulation used in the release studies.

The cumulative release ($\omega_{\text{cum}\;\text{released},i}$) was calculated as the sum of all the intervals:

(3) $$\omega_{\text{cum}\;\text{released},i}=\overset{i}{\underset{i=1}{\sum }}\omega_{\text{released},i}$$

Degradability of LYS-MSP-w in SLF (30 mg/L) was studied over 72 h at 37 °C at 150 RPM. To separate the solid fraction from the dissolution media, the sample aliquots were centrifuged (at 1500 G for 3 min) and washed 3 times with MilliQ-water. Finally, the washed centrifuged pellets were dried using a GeneVac EZ-2 Plus SpeedVac at 40 °C and 130 mbar. The SEM images of the dried pellets were analyzed as described in 2.4.4. and the silicon content in the dissolution media (supernatant) was analyzed by ICP-OES as described in Section 2.5.1. For the silicon content analysis, the unloaded MSP was also studied at the same concentration and conditions.

For understanding the remaining enzymatic activity of lysozyme after 24 h-release, the same method was applied. The lysozyme concentration in the supernatant was measured by UV-spectroscopy. An enzymatic activity assay for lysozyme from Sigma Aldrich (LY0100) was used to analyze the remaining activity of the protein according to the supplier procedure and at the recommended lysozyme concentrations. The remaining activity was calculated as the ratio between the lysozyme activity in the supernatant to activity of untreated lysozyme solution prepared from the kit at the same concentration.

#### 2.5.1. Silicon analysis with ICP-OES

Inductively Coupled Plasma Optical Emission spectroscopy (ICP-OES) measurements were performed using a Thermo Scientific iCap 6000. The silicon (Si) content was determined from the spectral intensities for Si at the wavelengths 2124, 2516 and 2881 Å. The Si concentration at a given time point represents the average of three repeat measurements performed on three independent repeat sample solutions. The percentage of Si dissolution is calculated by dividing the measured Si concentration (in mg/L or ppm) by the initial theoretical mass of Si. The initial theoretical mass of Si is calculated from the weighed mass of the particles added to the solution multiplied by the ratio of the molecular weights of Si (28.08 g/mol) and silica (SiO_2_; 80.08 g/mol). The time at which 50% Si dissolution occurs, denoted as the T_50%_, was interpolated from the linear region of the plot of the percentage of Si versus time graph by interpolating the curve in the linear lower time point region (0–8 h). It is acknowledged that the calculation of Si dissolved assumes all the sample mass consists of SiO_2_ and neglects any weight from surface groups, such as silanols. It is known from the literature that the degree of condensation does not have to be dramatic to result in 10–20% differences in the effective Si-loadings in the experiments (Braun et al., 2016). However, the production process of the MSP used in this study is designed to completely re-hydroxylate silica surface, as demonstrated in our previous works (Valetti et al., 2017; Angiolini et al., 2018). It is important to note that the Si dissolution of unloaded MSP were performed on filtered samples using 25 mm, 0.2 µm polyethersulfone (PES), whilst for LYS-MSP-w it was necessary to centrifuge the samples as described above to avoid obstruction of the filter with the sample and lose of measurable Si. The analysis was performed by triplicate.

### 2.6. Aerodynamic particle size distribution (APSD)

All APSD measurements were conducted using an ICOone® dry powder inhaler (Lastow & Arvidsson, 2015) (Figure 5(b)). The ICOone® is a single-dose, medium resistance ultralow-cost inhaler. It is injection molded in one piece and with the formulation protected by an Al-foil. The inhaler design features a cavity that can contain approximately 100 mm^3^ of powder. Each inhaler was filled with 10 mg of freeze-dried formulation (LYS-MSP). Using the Next Generation Impactor (NGI), the loaded dose was aerosolised at 64 L/min at pressure drop of 4 kPa until a total of 4 L of air was drawn through, conditions similar to those reported in literature (Buttini et al., 2016; van der Zwaan et al., 2024). A coating solution of 13% v/v Brij® 35 and 87% v/v glycerol in ethanol was used in each of the NGI stage cups to minimize particle bounce during the aerosolization. The aerosolized fractions were subsequently extracted from the device, inlet and adaptor, a pre-separator and different NGI stages using 15 mL of mixture of PBS buffer (150 mM, pH = 7.4) and internal standard (2.5 µg/mL of methyl-4-hydroxybenzoate). This internal standard was used to account for any losses during the recovery process. The lysozyme content of fraction was analyzed by high performance liquid chromatography (see Section 2.7). The fine particle fraction (FPF) refers to the portion of the drug contained within particles smaller than 5 µm relative to the total emitted dose. To calculate the mass median aerodynamic diameter (MMAD), the probability of mass against the natural logarithm of particle diameter was plotted to determine the particle size at 50% of the cumulative distribution (MMAD). Furthermore, the geometrical size distribution (GSD) was determined by calculating the range between the 84.13% and 15.87% cumulative distribution points.

### 2.7. High performance liquid chromatography (HPLC)

After extraction from the device, the samples were sonicated for 2 h to release the LYS (at room temperature), centrifuged at 3000 G for 10 min to remove MSP, and then the supernatant filtered with filter a Millex LG unit (low protein binding hydrophilic PTFE 0.20 µm membrane). HPLC was performed using the Agilent 1100 HPLC system (model G1312A) equipped with a diode array UV/visible detector (model G1362B). For the lysozyme quantification, a gradient elution method was developed using a 3 × 50 mm column, 2.6 μm (Kinetex® PS C18 column 100 A, Phenomenex), which consisted of a mixing of two mobile phases A and B with a flow rate of 0.8 mL/min. Mobile phase A consisted of HPLC MilliQ-water quality water with 0.1% v/v trifluoroacetic acid and mobile phase B was acetonitrile with 0.1% v/v trifluoroacetic acid. A detection wavelength of 280 nm was used, and the injection volume was 30 µL. The LOQ was 8.49 µg/mL.

### 2.8. Mithocondrial activity test (MTT)

Potential toxic effects on lung epithelium from the empty carrier (i.e. unloaded MSP) were investigated by evaluating the metabolic activity of Calu-3 cells. A mitochondrial activity test was performed based on the activity of the cytoplasmic NADPH dependent oxidoreductases by using the yellow tetrazolium salt 3-(4,5-dimethylthiazol-2-yl)-2,5-diphenyltetrazolium bromide (MTT) (Alfa Aesar). The MTT assay was chosen as a validated method for testing the cytotoxicity of EB in agreement with the OECD guidelines (OECD, 2017). Bronchial epithelial Calu-3 cell lines were obtained from American Type Culture Collection (ATCC) (Manassas, VA, USA), maintained at 37 °C and 5% CO_2_ and cultured in advanced minimal essential medium (AMEM) supplemented with 4 mM glutaMAX^TM^, 2.5% v/v FBS, 1 IU/mL penicillin and 1 μg/mL streptomycin. Exponentially growing cells were seeded at 1 × 10^6^ cells in 100 μL culture medium per well in 96-well plates and preincubated at 37 °C and 5% v/v CO_2_ for 24 h. The cells were incubated with eight different concentrations of MSP in PBS, ranging from 7.8 μg/mL to 1.0 mg/mL for 4 h at 37 °C. As negative control, the cells were treated with culture medium and PBS at pH 7.4 (i.e. untreated cells). As a positive control (i.e. toxic agent), the cells were treated with 10% w/v of benzalkonium chloride in PBS (Ihekwereme et al., 2014). MTT reagent was incubated with final concentration 0.5 mg/mL for 2h at 37 °C. The MTT solution was removed carefully and 100 μL of DMSO was added to solubilize the formazan crystals. Absorbance was measured at a wavelength of 570 nm using a Tecan Safire microplate reader (Florida, USA). The absorbance interference from cells was removed by subtracting the absorbance from untreated cells. The percentage of viable cells was calculated by the ratio of the average absorbance of MSP treated cells to untreated cells. This method allowed for eight replicates per concentration point. The fitting of dose-response curve follows previous report in literature (Hebbink et al., 2022) with a four-parameter logistic equation.

## 3. Results and discussion

### 3.1. Lysozyme loading capacity in disordered mesoporous silica particles

**Figure 1.**
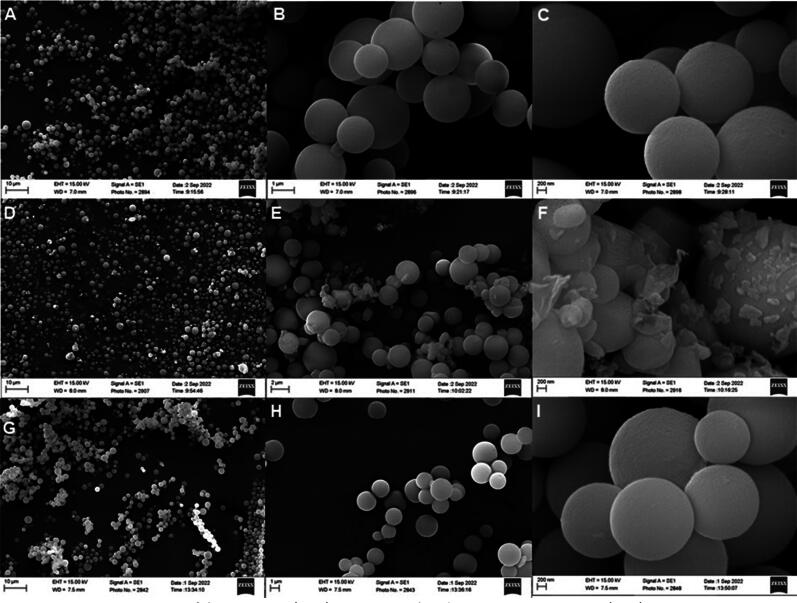
SEM images of bare MSP (A–C), LYS-MSP loading in 50 mM PBS (D–F), LYS-MSP-w loading in 50 mM with washing step (G–I).

**Figure 2.**
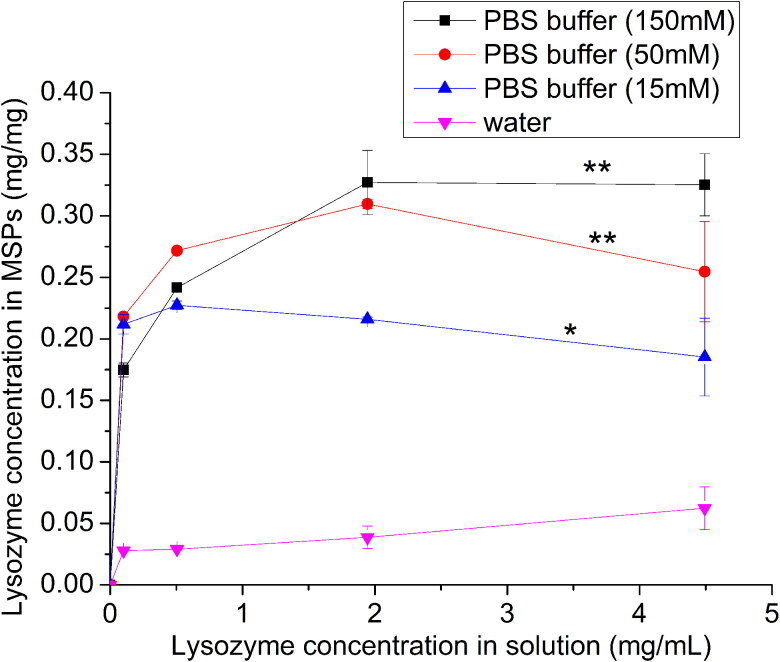
Adsorption isotherm at 25 °C of LYS on MSP at different ionic strengths of PBS at pH 7.4 and in pure water. Data showed as mean ± standard deviation (*n* = 3). Two ways anova test was performed and tukey test. Statistically significant difference is marked by *; *α* = 0.05 and **; *α* = 0.01 (t-student).

SEM images confirm the size and spherical shape of the mesoporous silica particles (MSP) (Figure 1(A–C)). The particle size distribution resulted to be 2.43 ± 0.13 µm as previously reported in our recent work combining image processing of electron microscopy and Elzone (coulter principle) analysis (Campos Pacheco et al., 2024). This is the desirable particles size range for inhalation delivery for dry powder inhalation (DPI) formulations (Liang et al., 2020). After loading of LYS, the surface appear to be uneven at 50,000 magnification (Figure 1(C)), likely due to the (sol-gel) synthetic method (Chunfang Zhou et al., 2018). On the other hand, proteins are versatile molecules that can interact with several surfaces due to its amphipathic nature, facilitating both electrostatic and hydrophobic interactions. For the former, pH and ionic strength primarily play an important role on electrostatic interactions and accordingly on adsorption. Figure 2 shows the absorption profile of LYS onto MSP using phosphate buffer saline (PBS), that has been previously shown to be a good loading media for silica particles (Cugia et al., 2016). Regarding the loading capacity, that it is expressed as mg of LYS per mg of MSP, a plateau state was reached around 0.3 mg of LYS per mg of MSP, which is in accordance with previous results (Kao et al., 2014). At this pH (i.e. pH 7.4), LYS is positively charged since below its isoelectric points (i.e. pI = 10.4) showing a Z-potential of +11.92 mV (0.3 mg/mL solution in PBS buffer 50 mM). While silica particles are negatively charged as we showed previously in a titration study using similar type of disordered MSP (Valetti et al., 2017). Thus, the selected pH provides the conditions that increase LYS-silica interactions, making possible to achieve loading. Vinu and collaborators similarly reported a good loading capacity between pH 6 and 10 (from 0.20 to 0.40 g/g respectively), which could be well described by the Langmuir model (Vinu et al., 2004). Our data also suggested that the adsorption follows Langmuir adsorption kinetics (see Supplementary Information, Figure S1).

The adsorption profiles also show that a higher ionic strength increases the adsorbed amount of LYS, especially in the region of high protein concentration (i.e. >2 mg/mL). The influence of ionic strength in the protein absorption profile has been already reported using colloidal (Meissner et al., 2015) and ordered porous silica SBA-15 (Steri et al., 2013), showing loading range from 0.25 to 0.95 mg/mg (Bhattacharyya et al., 2010; dos Santos et al., 2013; Steri et al., 2013; Moerz & Huber, 2015). Here, our data shows that this observation is valid also for disordered mesoporous. Meissner et al., reported that at pH < pI (i.e. pH > 7.4) the presence of salts increased the LYS adsorption to silica at rather high protein concentrations, likely due to a electrostatic screening that reduces repulsive electrostatic interactions between protein molecules in the adsorbed layer (Meissner et al., 2015). The presence of salt ions may affect not only protein-protein, but also protein – silica interactions. In particular, ionic strength can influence the dissociation constants and the effective surface charges of the silica surface. In fact, between pH 7–9, the magnitude of the MSP charge increases with increasing ionic strength, as shown in a previous study from some of the present authors (Valetti et al., 2017). This phenomenon was linked to the increased electrostatic screening (decreasing the Debye length), which reduces the free energy cost of charging the particle, permitting a higher surface charge. This increase in negative charge on the silica surface thus promotes adsorption of the positively charged LYS molecules. On the other hand, increased ionic strength may screen lysozyme – silica interactions and thus reduce adsorption. Nonetheless, since the maximum adsorption of LYS increased from 0.041 mg/mg in water to 0.35 mg/mg in 150 mM PBS. Overall, it is clear that the ionic strength causes the increase of protein adsorption. Nonetheless, for the loading process of lysozyme into MSP for DPI formulation preparation we selected to use ionic strength 50 mM with less salt amount.

After the LYS was adsorbed, the LYS-MSP formulations were freeze-dried to remove any residual water and scanning electron microscopy (SEM) and thermal analysis subsequently conducted to assess the obtained dry powder’s morphology and chemical composition. The SEM images in Figure 1 show the morphology of the LYS-MSP formulations after freeze-drying to consist of nano-sized agglomerates with irregular shape between MSP. These agglomerates are likely to be residual salts from the buffer (i.e. potassium and sodium phosphate, chloride salts) and potentially non-encapsulated lysozyme absorbed on the MSP’s outer surface. Excess salt content in DPI formulations is not desirable as is likely to deteriorate of aerodynamic properties. In fact, here, the aerodynamic distribution of LYS-MSP (without washing) was found to be inconsistent (see Supplementary Information, Figure S6), probably due to the inhomogeneity of the powder. Of interest, after addition of a washing step (LYS-MSP-w) (Figure 1(G–I)) no such residual salts or excess LYS was observed, instead the DPI formulation resembled that of the unloaded MSP, both at low and high magnifications.

To determine the nature of the observed residuals, thermogravimetric analysis (TGA) was performed (Figure S3 and Table 2). The interpretation of the curves of the freeze-dried formulations was based on the thermal decomposition profiles of the unloaded MSP, PBS and LYS (see Supplementary Information, Figures S2 and S3). The water content (mass loss from 25° to 100 °C) was slightly lower (2.7% vs. 3.5%) for the formulations with lower PBS molarity since the buffer PBS component salts have crystal hydrates. The water content was also decreased after the washing step (i.e. LYS-MSP-w). Moreover, in the LYS-MSP-w, the decomposition curve of PBS (temperature range 750–1000 °C) was completely absent, suggesting that the washing step was successful to remove the salt ions even inside the particlés pores. The lysozyme content (temperature range 200–800 °C, see Supplementary Information, Figure S2) in the LYS-MSP-w was lower than in the washed ones, suggesting that 6.4% and 11.8% of the LYS is washed away from the loading in 50 mM or 150 mM PBS, respectively (Table 2). This confirms also that part of nano-sized agglomerates between the spherical particles of MSP observed at SEM was not encapsulated LYS.

**Figure 3.**
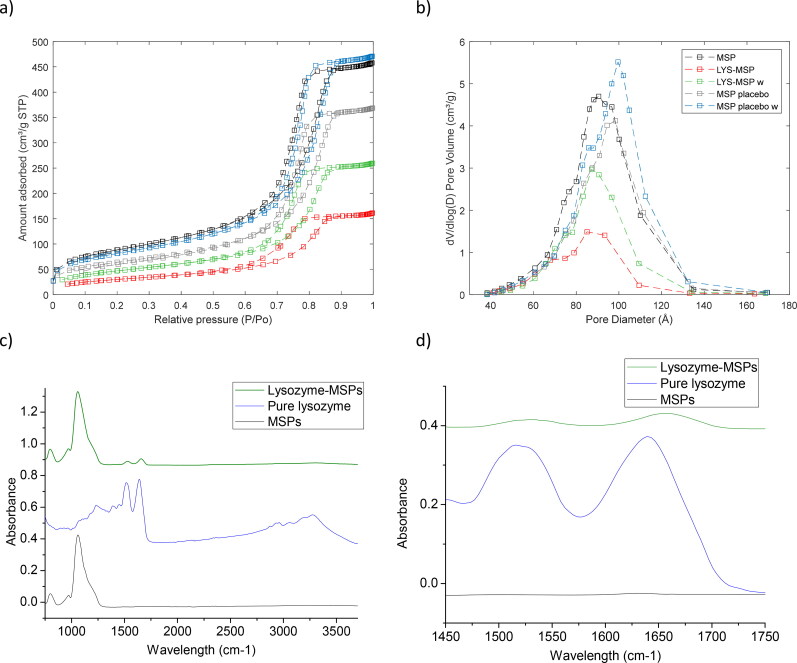
(a) Nitrogen adsorption/desorption isotherms and (b) pore size distribution of MSP, LYS-MSP, LYS-MSP-w, MSP-placebo (i.e. MSP-PBS) and MSP-placebo-w (i.e. MSP-PBS after washing step), 50 mM PBS buffer was used. (c) ATR-FTIR spectra of MSPs, pure LYS and LYS-MSPs in the range 750–3700 cm^−1^. (d) ATR-FTIR in the range of 1450–1750 cm^−1^ highlighting the amide group of LYS.

**Table 2.** TGA analysis of freeze-dried formulations.

	LYS-MSP 50 mM PBS	LYS-MSP-w 50 mM PBS	LYS-MSP 150 mM	LYS-MSP-w 150 mM PBS
Water (weight loss in wt % between 25°- 100 °C)	2.7 ± 0.5	2.3 ± 0.4	3.5 ± 0.4	2.9 ± 0.2
Lysozyme^a^ (weight loss in wt % between 200 °C −800 °C)	14.2 ± 0.2	7.8 ± 0.4	19.9 ± 0.7	8.1 ± 0.3
Salts (weight loss in wt % between 800 °C −1000 °C)	21.0 ± 0.1	–	47.8 ± 0.8	–
MSP^b^ (the rest)	62.1 ± 0.5	89.9 ± 0.5	28.8 ± 0.9	89 ± 0.5

^a^ Values for lysozyme content contain correction on amount of silanol group (2.5%) condensation reaction occurring between 200 °C −800 °C.

**Table 3.** Characteristics of the aerodynamic properties of LYS-MSP-w.

Aerodynamic properties	Values
FPF (%)	70.32 ± 1.38
MMAD (µm)	1.64 ± 0.06
GSD	1.69 ± 0.01
Retention in the inhaler (%)	9.55 ± 1.34

### 3.2. Nanoconfinement of lysozyme in the disordered mesoporous structure

MSP were synthesized with large pore size for the encapsulation of lysozyme to the pores (Figure 3(b)). MSP have broad pore size distribution from 30 to 125 Å with an average pore diameter of 80.5 Å (Table 1; Figure 3) determined from BET, differs from the uniform distribution of ordered mesoporous silica materials, such MCM-41 or SBA-15 (Shen et al., 2011). For successful loading of protein inside the MSP, it is generally suggested to have pores twice bigger than the size of the molecule. In fact, lysozyme molecules in pure water have an ellipsoid shape with radii of 27.2 × 15.5 Å according to small angle X-ray scattering (Phan-Xuan et al., 2020), suggests a maximum molecular diameter of 54 Å. This is in agreement with the reported radius of 21 Å in phosphate saline buffer evaluated with dynamic light scattering (Falke et al., 2018). Therefore, according to steric considerations lysozyme could enter inside the pores. To determines if the LYS adsorption occurs at the external and/or internal surface of particles, the pore filling of MSP was evaluated by N_2_ sorption/desorption. The N_2_ sorption/desorption of unloaded MSP shows an IV-type isotherm (Figure 3(a)), that is the typical isotherm representing the multilayer adsorption, together with capillary condensation for mesoporous material. After LYS loading, the characteristic IV-shape remains, but with a significant reduction of adsorbed nitrogen, suggesting that the mesoporous structure of the MSP is retained, but that the protein is encapsulated inside the particle pores, reducing the available pore volume of the MSP. In contrast, the presence of non-encapsulated LYS at the surface of the particles could instead block the pore entrance. However, since the N_2_ adsorption/desorption have the same IV shape after LYS adsorption, such blockage of the outer pores is expected to be considered to be minimal. Compared to an unloaded MSP, the LYS-MSP have a reduced surface area and surface area of about 65% (Figure 3; Table 1). It is also interesting to note that pore filling primarily appears to occur for pores with diameters between 70 and 125 Å (Figure 3). Such ­preferential adsorption of the LYS into the larger MSP pores could suggest some steric hindrance of the LYS (i.e. ­maximum diameter of 54 Å) prevents adsorption into the smaller pores.

**Table 1.** Pore structure of MSP, LYS-MSP and placebo formulations.

Sample	Surface area^a^ (m^2^/g)	Pore volume^b^ (cm^3^/g)	Average pore diameter^c^ (Å)
MSP	312	0.70	80.5
LYS MSP	108	0.25	73.5
LYS-MSP-w	171	0.40	78.5
MSP placebo	228	0.64	82.2
MSP placebo-w	289	0.72	85.4

^a^ BET surface area. ^b^at a relative pressure (P/P_0_) > 0.98. ^c^BJH average pore diameter.

It is known that the PBS alone can fill the pores of the MSP, notably reducing the pore volume (Garcia et al., 2016). To assess if PBS was also present in the pores, N_2_ sorption/desorption was conducted on of MSP-placebo that had been incubated only with PBS and the dependence of the PBS ionic strength on the sorption properties of the placebo formulations investigated (see Supplementary Information, Figure S4). Our data agree with the previous observations that PBS does adsorb inside the pores (Garcia et al., 2016), but of interest the surface area and pore volume reduction in MSP-placebo are only 26% and 8%, respectively. Moreover, previous reports on ordered silica particles showed similar differences in pore size dimension after LYS loading (Bhattacharyya et al., 2010; Steri et al., 2013) supporting that our data indicate that in the LYS-MSP, LYS is the main loading fraction. After one washing step with water, the particles (i.e. LYS-MSP-w) showed an intermediate reduction in surface area (i.e. 45%) and pore volume (i.e. 43%), confirming the previous indications (Figure 1 and Table 2) that part of lysozyme and PBS are rinsed during the washing step. Another observation was that, after washing, the porosity of MSP-placebo is independent of the initial amount of PBS loaded in the particle (see Supplementary Information, Figure S4), suggesting that the removal of the buffer is effective, independent of the ionic strength of the loading media. However, after the washing step, the pore size distribution appears to narrow and shift toward larger pore sizes when compared to the unloaded MSP, which could suggest some initial dissolution of the particle’s matrix occurs during washing (Braun et al., 2016). The confirmation of lysozyme adsorption into mesoporous silica particles was also supported by ATR-FTIR spectroscopy (Figure 3(c,d)). The infrared signals of lysozyme are characterized by the carbonyl group that belong to the peptide bonds. Mainly lysozyme presents the bands amide I and II. Amide I typically appears in the region of 1600–1700 cm^−1^, which is associated to C = O stretching. Amide II, which is a combination of N–H bending and C–N stretching, typically appears approximately in the region of 1450–1550 cm^−1^ (Osta et al., 2020; Velk et al., 2023). Figure 3(c,d) shows these bands for pure lysozyme and for the dry powder LYS-MSPs, providing complementary evidence of its sorption within mesoporous silica particles. Moreover, LYS-MSPs also exhibit strong Si-OH signals around 1000–1250 cm^−1^.

Altogether, the N_2_ adsorption/desorption and FT-IR data indicate that LYS fills the MSP pores and preferentially the larger pores fractions. Moreover, the majority of the filled (encapsulated) volume is LYS, rather than any residual buffer salts. The observed decrease of pore diameter in loaded samples is in agreement with previous reports for small molecules (Shen et al., 2011) and proteins encapsulated in ordered silica materials (Garcia et al., 2016).

The maximum amount of lysozyme that can be loaded into the pores of the MSP can be estimated by the following equation:

(4) $$m_{\text{lys}}=\rho_{\text{lys}}\cdot \varphi \cdot V_{\text{pore MSP}}$$

if the following assumptions can be made: all pores are filled by protein, protein is an ellipsoid, the packing density of ellipsoids is φ=0.74 (Donev et al., 2004) and the lysozyme density $\rho_{\text{lys}}=1.4\;g/{\text{cm}}^{3}$.

For the MSP used in this study, this equates to a maximum capacity of 0.68 g of lysozyme per 1 g of unloaded MSP. Although, it is likely that the maximum amount is not possible to achieve practically due to steric hindrance of lysozyme (see discussion above).

Alternatively, it is also possible to calculate the achieved loading capacity from the reduction of pore volume measured by N_2_ adsorption/desorption considering that:

(5) $$V_{\text{pore loaded MSP}}=\frac{V_{\text{pore MSP}}-V_{\text{lys}}}{m_{\text{MSP}}+m_{\text{lys}}}\;$$

(6) (for 1 g of MSP) $V_{\text{pore loaded MSP}}=\frac{V_{\text{pore MSP}}-V_{\text{lys}}}{1+m_{\text{lys}}}=\frac{V_{\text{pore MSP}}-\frac{m_{\text{lys}}}{\rho_{\text{lys}}}}{1+m_{\text{lys}}}$

For the unloaded MSP used in this study, Eq. 6 results in a total of $m_{\text{lys}}$=0.21 g per g of MSP. This value is close to the loading capacity of LYS-MSP obtained in lysozyme sorption experiment in Section section 3.1 for 50 mM PBS (Figure 2), i.e. 0.35 mg of LYS per mg of MSP. Therefore, it is likely the difference between these two values is due LYS losses during the freeze-drying process.

**Figure 4.**
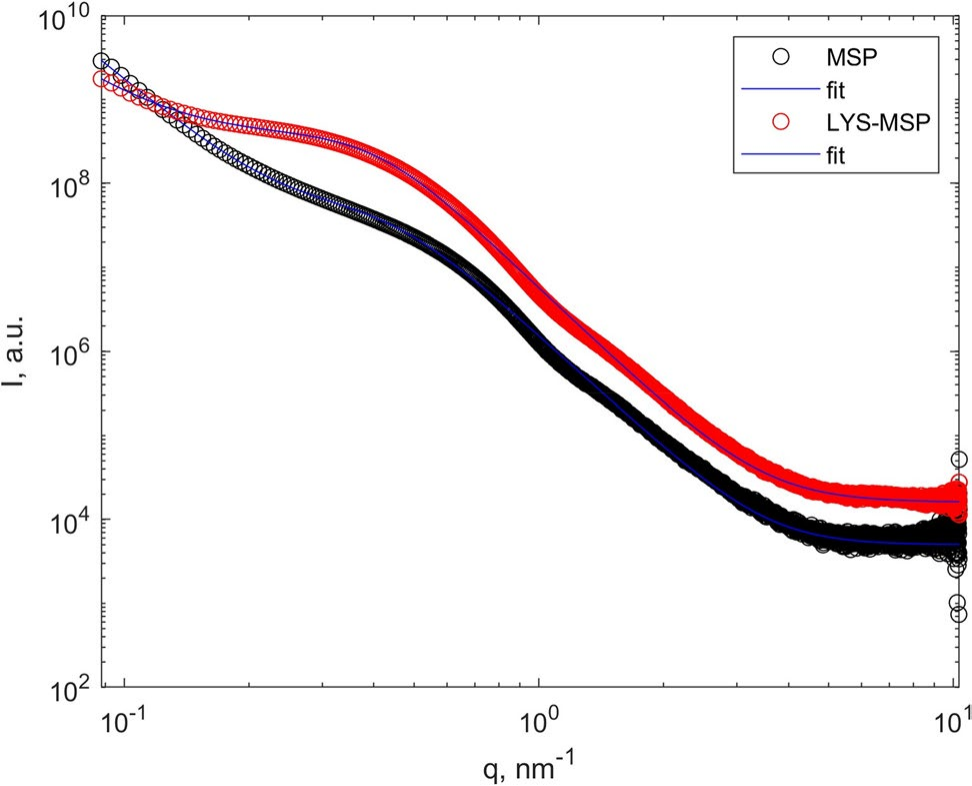
SAXS curves of bare MSP (black) and after lysozyme loading in 50 mM PBS buffer and freeze-drying without washing (LYS-MSP, red) along with the best fit (blue lines) using the correlation length model. The fitting results are reported in Table S1.

As complementary technique to confirm the presence of LYS inside mesopores, we investigated small angle X-ray scattering (SAXS) to understand the internal porosity of the particles (Gommes et al., 2016). Given the lowest accessible q for synchrotron SAXS as 0.05 nm^−1^ (i.e. corresponding roughly to a maximum of 120 nm), this analysis meant to study the internal pores of the particle rather than the particles itself. An overview of the SAXS patterns of unloaded MSP and LYS-MSP are presented in Figure 4. The scattering intensity of LYS-MSP is higher than the unloaded MSP. Scattering intensity of studied material depends on the scattering intensity of the chemical composition and density. The loaded material has higher density and another chemical composition (presents of LYS and buffer salt) that leads to higher scattering intensity. The SAXS patterns of MSP and LYS-MSP display a broad peak at lower q-values that corresponds with a broad disordered pore structure. The peak slightly shifts to lower q-values (from 0.6 to 0.5 nm^−1^) for the loaded LYS-MSP, which suggests a change to the MSP pore structure.

The unloaded MSP scattering curve exhibits a Porod power law dependence (Eq. 1) at *q* < 1 nm^−1^ with a slope of ∼ q^4^ (Figure 4; Table S1), which is related to the air-solid interface of MSP (Roe, 2000). The slope decreases to q^3.2^ for the LYS-MSP material (Figure 4; Table S1), consistent with fractal surface (Roe, 2000). In the original application of the employed fitting model (Eq. 1), the fitting parameter ξ describes the correlation length for the polymer chains. Instead, here, we suggest that in case of the disordered porous silica material, this parameter can be related to the average wall thickness. The parameter ξ in MSP is 22.2 Å, while in loaded material is 24.3 Å. This change thus suggests an increase of the wall thickness upon lysozyme and buffer sorption, due to material deposition inside the pore. The increase of wall thickness was observed for the DPI formulation after washing step (i.e. LYS-MSP-w) using a laboratory X-ray source (see Supplementary Information, Figure S5). The changing of the wall thickness of silica material with and without lysozyme has been also previously reported for silica − lysozyme composites (Bruno et al., 2022).

In summary, N_2_ adsorption/desorption and SAXS studies confirmed that: lysozyme is located within the pores of the disordered MSP; an additional washing step with water completely removes any (external) excess salts or lysozyme, as shown by TGA and SEM; and a good portion of LYS is retained after washing (from 7.8% to 8.1%; Table 2).

### 3.3. Aerodynamic properties and lung deposition

**Figure 5.**
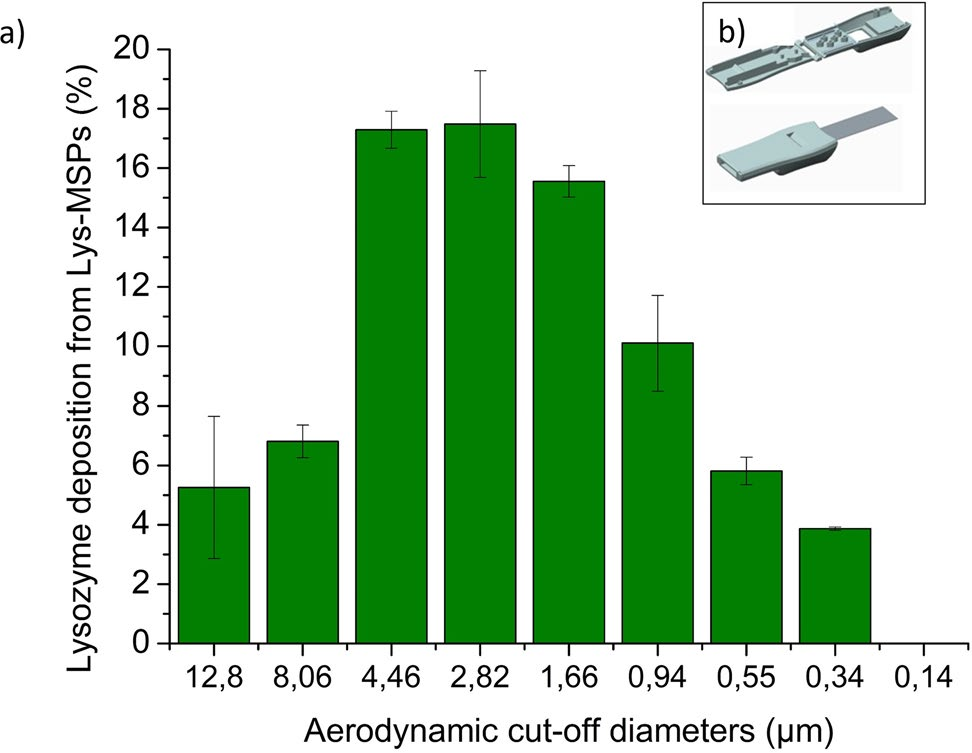
(a) Aerodynamic distribution using next generation impactor (NGI) of LYS-MSP-w (loading in 50 mM PBS) from NGI and aerodynamic properties, the FPF (%) is based on the total emitted dose. Data are presented as means ± SD (*n* = 3). (b) ICOone inhaler. Open (above) and closed (below) with Al-foil.

The aerodynamic distribution profile of the LYS-MSP-w DPI formulation was evaluated by use of the next generator impactor (NGI). Figure 5(a) shows the aerodynamic distribution of LYS-MSP-w, after actuation from ICOone® dry powder inhaler (Figure 5(b)). Device retention was 9.55 ± 1.34 (Table 3), and the delivered dose was 90.5 ± 0.9%. The Fine Particle Fraction (FPF, for a diameter <5 µm) was 70.32% (Table 3), which is in the range of other literature reported DPI protein formulations (i.e. with lysozyme, ciprofloxacin) that have FPF of 60 − 80% (Ogáin et al., 2011; Ferrati et al., 2018; Adhikari et al., 2022). This result indicates that disordered MSP are suitable DPI carriers for biologics, without the need of additional excipients. It is important to note that, in contrast, aerodynamic assessment of the non-washed formulation (LYS-MSP) resulted in a lower FPF (i.e. 65.6% Figure S6), emphasizing the importance of the post-adsorption washing step for achieving increase LYS deposition in the lung, despite reducing its content in the DPI formulation. In a recent study the in vivo assessment of disordered MSP has been successfully demonstrated in the lung of rats following inhalation of controlled doses (Pilkington et al., 2023). A target dose (∼500 μg) was delivered to intubated rat lungs using a PreciseInhale® (Inhalation Sciences) dosing system (Fioni et al., 2018) and the clearance of the deposited particles determined over a 12-h period (using tissue digestion and a plasma emission spectroscopy method) providing the direct evidence of the MSP ability to be successfully delivered to the lungs in an animal model.

### 3.4. Carrier degradability, lysozyme release and activity in simulated lung fluid

**Figure 6.**
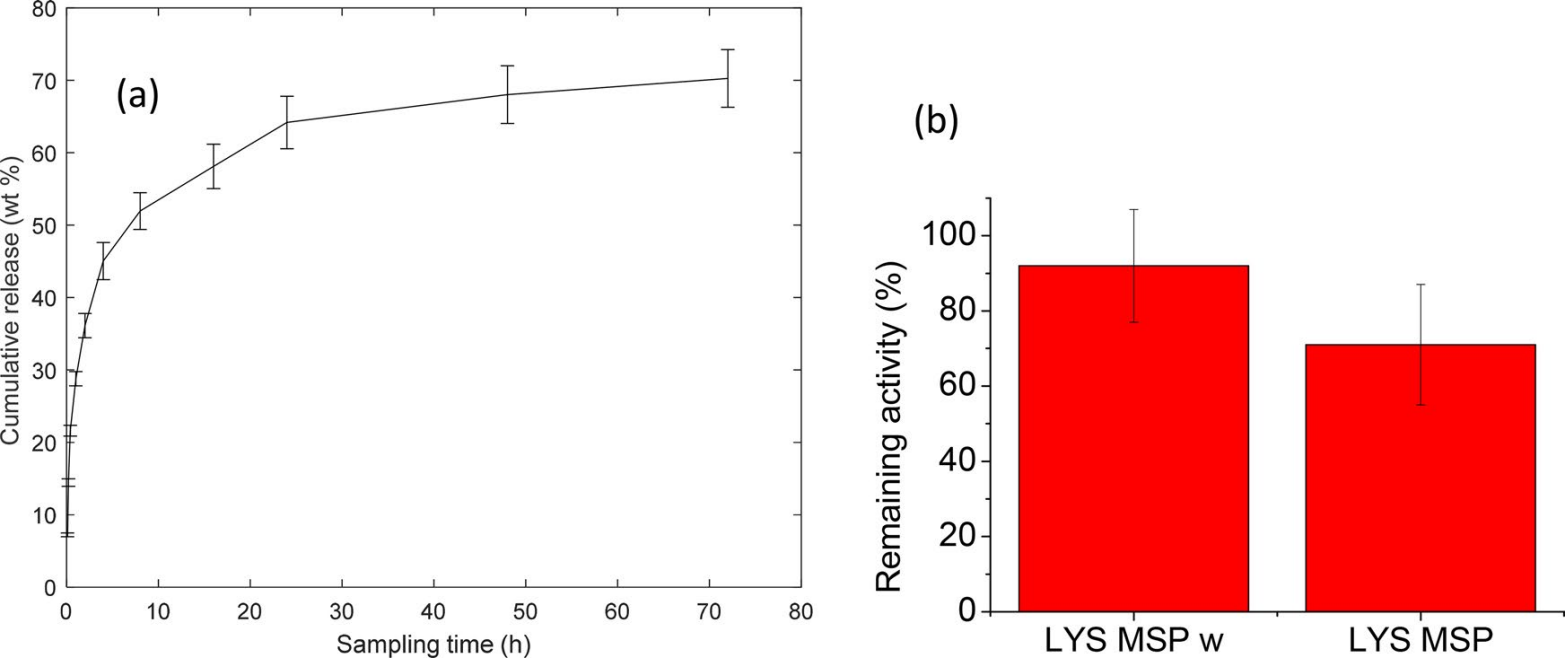
(a) Cumulative release of lysozyme from LYS-MSP-w (loaded in 50 mM PBS) in simulated lung fluid at 37 °C. (b) Remaining lysozyme activity after release in simulated lung fluid. Data are shown as mean ± standard deviation (*n* = 3). According to T-test the data set is significantly different (*α* = 0.05).

After deposition in the lung, the encapsulated protein needs to be released in the lung lining fluid. To better understand the release kinetics of LYS in the lung lining, the release of LYS was evaluated for the LYS-MSP-w formulation in Gamblés solution, which is a commonly used simulated lung fluid (SLF) (Marques et al., 2011). Figure 6(a) shows that the majority of the encapsulated LYS is released in the first 24 h (70.30 ± 3.99%). The observed sustained release kinetic is likely due to the slow protein slower diffusion of macromolecule from the MSP. In comparison with ordered mesoporous silica materials, such SBA-15 and nano fibers (Steri et al., 2013; Henry et al., 2017), our data show that LYS is released from disordered MSP with a slower kinetics (i.e. plateau reached after 24 h vs 10 h) but in a higher extent (i.e. total LYS released 70% vs 40%). Therefore, this suggests that disordered mesoporous, holding a broad pore distribution, will offer a higher protein dose in an extent period which could be more appropriate to reduce the dose frequency. Moreover, the electrostatic attraction between the positive charged lysozyme (pI ∼ 11) and negatively charged MSP at pH 7.4 could play an important role in hindering the LYS release. However, this is less likely to be the case for the majority of the therapeutic proteins, which are more commonly negatively charged at physiological pH of lungs. Therefore, for other type of proteins the release in SLF from the MSP could be faster than the particle internalization in cells. Moreover, a recent report showed that MSPs escape the lysosomal degradation by causing rupture of the endosomal/lysosomal membrane by generating ROS, showing promises to deliver active biologics molecules (Hong et al., 2020).

An essential aspect to consider in the development of a therapeutic protein formulation is the biological activity of the delivered protein when released. For the washed formulation, LYS-MSP-w, the enzymatic activity of the LYS released in SLF over 24 h was 92% and significantly higher than that of the non-washed (LYS-MSP) formulation, which had an activity of 70%. Earlier it has shown that lysozyme remains enzymatically active after being released from silica nanofibers (Henry et al., 2017) and ordered MSP (Kao et al., 2014) in different buffers. Our work here in addition demonstrates that lysozyme stays active after being released from disordered MSP in SLF that are relevant for pulmonary drug delivery. It is likely that the released LYS from the unwashed formulation (LYS-MSP) exhibits a lower activity because of freezing stresses caused by phosphate salts (Kolhe et al., 2010; Thorat & Suryanarayanan, 2019). This observation thus further corroborates the importance of removing the residual buffer before freeze-drying.

**Figure 7.**
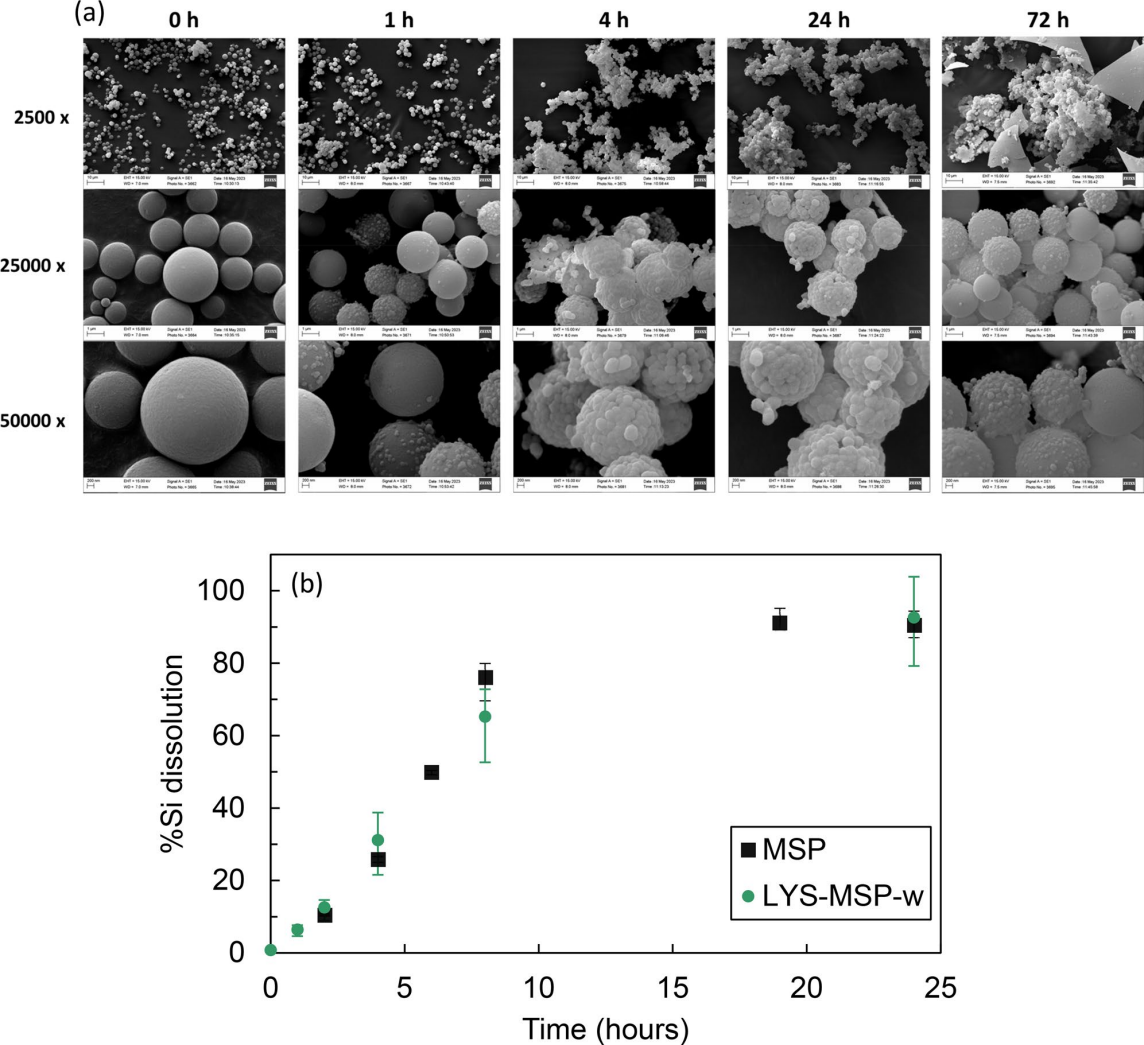
(a) SEM analysis of degraded LYS-MSP-w in simulated lung fluid with an initial dose of 30 mg/L. Different time samples (0 h, 1 h, 4 h, 24 and 72 h) were analyzed at 2500, 25,000 and 50,000 x. (b) Dissolution kinetics of Si from LYS-MSP-w and MSP control (30 mg/L) in SLF, presented as percentage of Si dissolved from the initial amount. Data are presented as the mean ± SD (*n = 3*).

Another important aspect of consideration in the development of a pulmonary drug delivery formulation, is the clearance of the MSP carrier from the lungs. After exposure to simulated lung fluid, the SEM images in Figure 7(a) indicate that the micron-sized carrier MSP particles in LYS-MSP-w underwent degradation within 4 h, forming aggregated nanoparticles in micron-sized structures, while recrystallization of lysozyme occurs (observed after 72 h as micron-sized debris). The drastic degradation of polyethyleneimine coated silica particles have been shown previously in PBS due to proton sponge effect of a polyethyleneimine (Choi & Kim, 2019). Our study shows a new observation for the degradation of uncoated disordered MSP into aggregates nanoparticles. The formation of these aggregated nanoparticles can be attributed to the sol-gel synthesis of MSP, which occurs through the condensation process of smaller nanoparticles. In contrast, an on-going work in our group shows that uncoated disordered MSP loaded with clofazimine, a small antibiotic molecule, degraded in SLF forming non-aggregated nanoparticles, offering a dual micro-nano carrier strategy to tackle resistant bacteria inside macrophages (Pacheco et al., 2024). Hence, the aggregation of the nanoparticles observed here is likely due to the presence of a stronger interaction between the silica surface and LYS protein that could contribute to aggregation of the degraded MSP nanoparticles (Lin et al., 2022).

Quantification of the silica dissolution of LYS-MSP-w in SLF is shown in Figure 7(b). The dissolution kinetics of Si exhibited a linear trend within the 0–8 h, enabling the determination of the time required for 50% dissolution (T_50%_) of the initial Si mass, which was found to be 6.2 h. Almost complete dissolution of the Si (93%) was achieved after 24 h and it is likely that the remaining 7% was lost during the recovery process, which involved centrifugation, washing and filtering (see Section 2.5.1). It is worth noting that the dissolution of the unloaded MSP showed a similar Si dissolution profile (Figure 7(b)), with a T_50%_ value of 5.9 h, indicating that the presence of lysozyme only does not significantly retard the silica dissolution process. The Si dissolution of unloaded particles is in agreement with our previous data at 37 °C T_50%_ value of 5.5 h (Pacheco et al., 2024). It is known that amorphous silica, such as the disordered MSPs studied here, dissolve in biological fluids to a soluble form of silicon dioxide, SiO_4_ tetrahedra. Additionally, the presence of electrolytes, particularly alkaline cations (Na^+^, K^+^, Mg^2+^) found in lung fluid, has demonstrated to increase the dissolution rate through the detachment of higher coordinated species, creating a periphery of reactive, lesser coordinated groups that enhance the surface energy (Dove et al., 2008). Moreover, the Si dissolution data for the LYS-MSP-w formulation suggests that a dry powder dose of 30 mg/L is recommended to achieve complete particle dissolution in lung fluid within 24 h. This can allow estimate a maximum single dose per day as frequency of dose considering the carrier biosafety as a primordial aspect, which can be of interest for the delivery of a protein with therapeutic effect. Yet, to fully assess the frequency of dose and safety profile, more comprehensive regulatory in vivo toxicity studies would be required.

### 3.5. Safety assessment of MSP on lung epithelium

**Figure 8.**
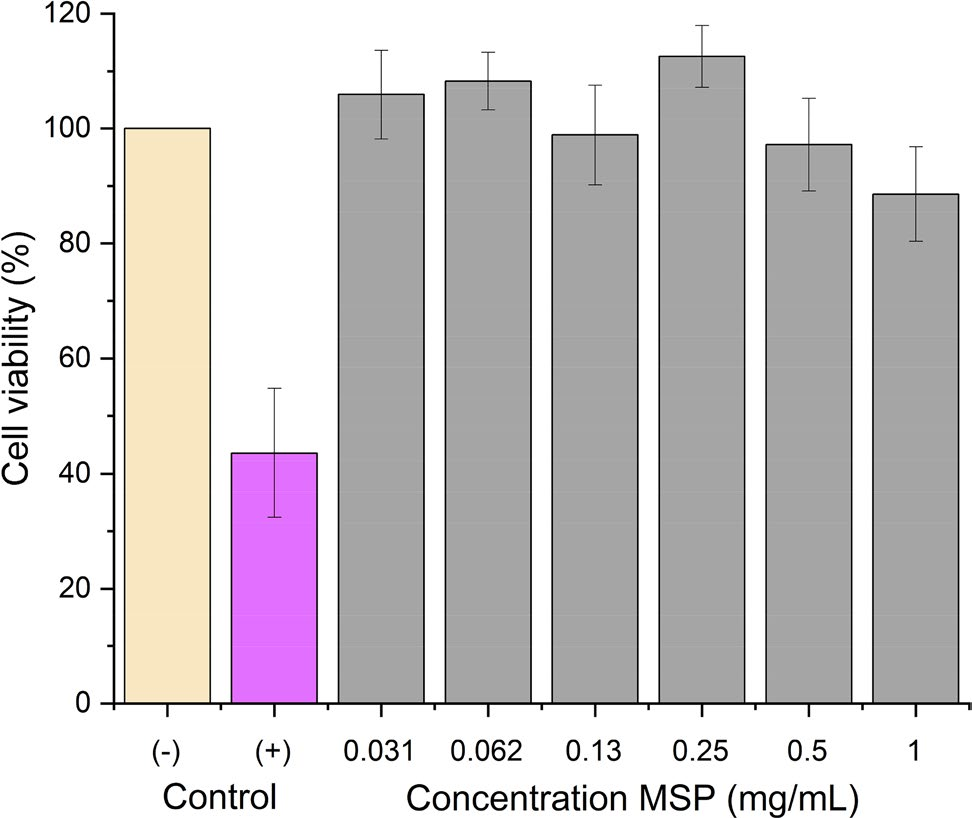
Cell viability at different bare MSP concentrations (i.e. without LYS, placebo formulation). The viability of bare MSP is normalized versus the untreated cells, i.e. treated with the same buffer used for suspension of particles (negative control), and versus a positive control (benzalkonium chloride 10%). The data are presented as the mean±SD (n=8)

The potential toxicity of the disordered mesoporous silica particles alone in the lungs is one of the most important issues which needs to be addressed to investigate the new drug carrier. In the present study, we conducted a mithocondrial activity test (MTT) as cell viability assay after incubation of bare MSPs (i.e. without LYS, placebo formulation). As for cells, we used the Calu-3 cells are derived from human bronchial epithelium and represent a broadly used and robust cell line due to their ability to form tight monolayer. After 4 h incubation, the cell viability was maintained up to an MSP concentration of 1 mg/mL at pH 7.4 (Figure 8), i.e. no toxic effect on lung epithelium was found (i.e. no statistical differences vs. the control, *α* = 0.05). Benzalkonium chloride (10%) was used as positive control since it is known to be irritant/toxic to airway cells (Ihekwereme et al., 2014). A recent report has concluded that different parameters may influence cytotoxicity results, such as physicochemical features of MSP (i.e. particle size, zeta-potential and surface functionalisation) (Ahmadi et al., 2022). Previously, it has showed before that MSP up to 500 nm were safe (concentration 10, 25 µg/mL)on Calu-3 cells using MTT assay (McCarthy et al., 2012). Here we have shown that also micro-sized, disordered (unloaded) MSP are nontoxic over a therapeutically relevant concentration range for lung therapies of 30 mg/L. These results are promising for a safe inhalation treatment and pave the way for further *in vivo* assessment of disordered MSP. Future directions should be aimed to address in vivo clearance of the MSP in animal model to understand the risk of potential chronic toxicity.

## 4. Conclusions

Here we present for the first time, to the best of our knowledge, a proof-of-concept of dry powder inhalation (DPI) formulation for protein lung delivery using disordered MSP as sole encapsulating excipient. Adsorption of lysozyme into the disordered MSP was demonstrated to increase with the ionic strength of the phosphate buffer loading media but did not change significantly change between 50 and 150 mM. A simple post-adsorption washing step was shown to be effective in removing residual buffer salts and unabsorbed protein, leading to a homogeneous freeze-dried powder. The presence of lysozyme was confirmed by TGA and FT-IR, while N_2_ sorption/desorption and SAXS analysis of the washed freeze-dried formulation revealed that lysozyme is encapsulated within the MSP nanopore structure. Aerosolisation of the freeze-dried formulation from a low-cost, single dose ICOone® inhaler, without addition of any other excipients, was demonstrated to result in an excellent aerodynamic distribution (i.e. FPF. = 70.32 ± 1.38%), with a relatively low device retention (9.55 ± 1.34%). Dissolution tests showed that the protein is slowly released in simulated lung fluid over 72 h and maintained its enzymatic activity (i.e. 92% of the initial value). Degradation of the lysozyme DPI formulation in simulated lung fluid was shown to occur within 4 h and produce micron-sized nanoparticle aggregates, likely due to the attractive interaction between the released lysozyme and degraded MSP nanoparticles. *In vitro* safety tests showed disordered MSP carrier to be non-toxic to the lung epithelium at and beyond therapeutically relevant concentrations. In a summary, this work presents disordered MSP as novel and effective DPI carrier for therapeutic proteins/antibodies/vaccines for pulmonary delivery.

## Supplementary Material

Supporting info.docx

## Data Availability

Data available within the article or its supplementary materials. Data can be shared upon request from sabrina.valetti@mau.se.
