# Supplementary material for: Disordered mesoporous silica particles: an emerging platform to deliver proteins to the lungs
Source: Drug Deliv. 2024 Jul 23;31(1):2381340. doi: 10.1080/10717544.2024.2381340 (PMC11268259; doi:10.1080/10717544.2024.2381340)
Supplement: Supporting info.docx [file IDRD_A_2381340_SM3007.docx]

**Disordered mesoporous silica particles: an emerging platform to deliver proteins to the lungs**

***Supporting Information***

Contents

[**Figure S1.** Fit of the adsorption experimental data employing the Langmuir model. 2](#_Toc141949262)

[**Figure S2.** Thermal decomposition of lysozyme obtained by TGA. 3](#_Toc141949263)

[**Figure S3.** TGA curves of thermal decomposition of PBS, bare MSP and formulations LYS-MSP and LYS-MSP-w, 50 mM PBS buffer was used for loading. 3](#_Toc141949264)

[**Figure S4.** N_2_ adsorption/desorption as a dependency of PBS ionic strength, pore size distribution (a,b) and sorption isotherms (c,d). 4](#_Toc141949265)

[**Figure S5.** SAXS curves of bare MSP (black) and after lysozyme loading in 50 mM PBS buffer with washing LYS MSP w (green). 4](#_Toc141949266)

[**Table S1.** Parameters extracted from the modeling of SAXS pattern in SAS view by correlation length model (Eq.1). Values in parentheses are standard deviations. 5](#_Toc141949267)

[**Figure S6.** Aerodynamic distribution using next generation impactor (NGI) of LYS-MSP. 5](#_Toc141949268)

## **Figure S1.** Fit of the adsorption experimental data employing the Langmuir model.

The amount of LYS adsorbed on the MSPs adsorbent was fitting with Langmuir isotherm model:

$$r=\frac{r_{0}\cdot K\cdot c_{lys in sol}}{1+K\cdot c_{lys in sol}}$$

Where $r$ is ration between adsorbed LYS at MSP $\frac{{mg}_{lys}}{{mg}_{MSP}}$ , $r_{0}$- maximum adsorbed LYS, $K$ constant of adsorption, $c_{lys in sol}$ concentration of LYS in solution.

|  | water | 15 mM | 50 mM | 150 mM |
| --- | --- | --- | --- | --- |
| $r_{0}$(mg/mg) | 0.037 | 0.22 | 0.32 | 0.34 |
| K constant of adsorption (ml/mg) | 15.63 | 16.41 | 19.64 | 8.15 |

## **Figure S2.** Thermal decomposition of lysozyme obtained by TGA.


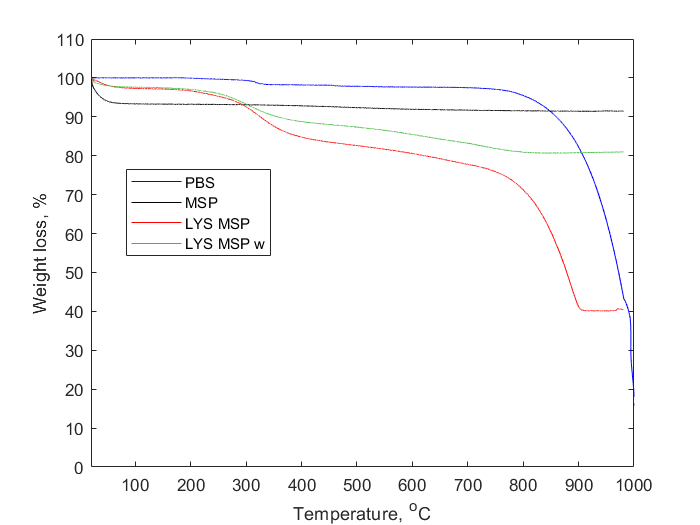


## **Figure S3.** TGA curves of thermal decomposition of PBS, bare MSP and formulations LYS-MSP and LYS-MSP-w, 50 mM PBS buffer was used for loading.

| a) | b) |
| --- | --- |
| 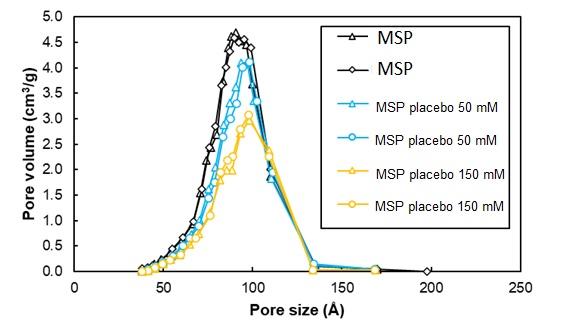 | 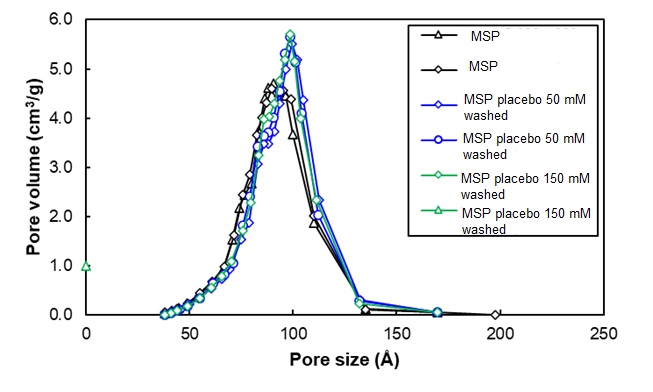 |
| c) | d) |
| 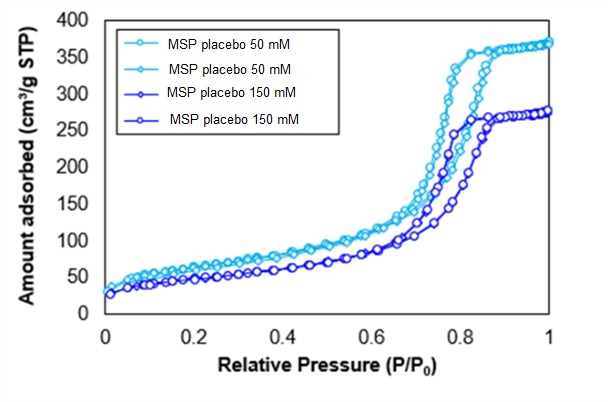 | 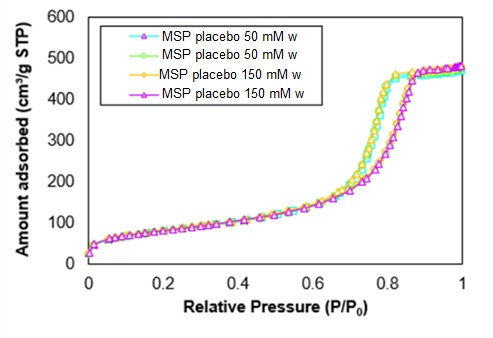 |

## **Figure S4.** N_2_ adsorption/desorption as a dependency of PBS ionic strength, pore size distribution (a,b) and sorption isotherms (c,d).


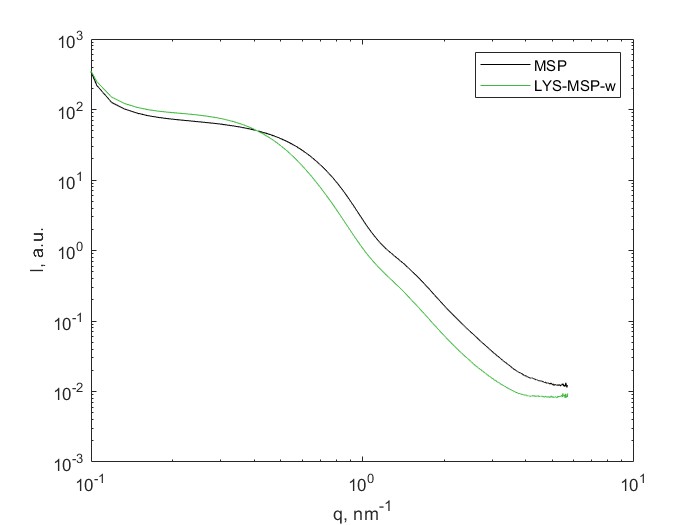


## **Figure S5.** SAXS curves of bare MSP (black) and after lysozyme loading in 50 mM PBS buffer with washing LYS-MSP-w (green).

Data collected at XEUSS 3.0 X-ray scattering instrument.

## **Table S1.** Parameters extracted from the modeling of SAXS pattern in SAS view by correlation length model (Eq.1). Values in parentheses are standard deviations.

|  | Synchrotron source | | Laboratory source |
| --- | --- | --- | --- |
| Parameter | MSP | LYS-MSP | LYS-MSP-w |
| Background | 5000 | 16000 | - |
| Lorenz scale (C) | 5.264e+07  (±3727.3) | 3.8787e+08  (11652) | 2.8123e+02  (152) |
| Porod  Scale (A) | 1.5772e+05  (±18.971) | 4.7386e+05  (140.1) | 23  (3) |
| Cor-length  $\boldsymbol{(\xi)}$ nm | 2.2225  (±7e-05) | 2.4317  (2.8247e-05) | 2.3889  (7.6312e-06) |
| Porod  Exp (n) | 4.0364  (±5.2568e-05) | 3.2813  (0.0001242) | 3.367  (0.004532) |
| Lorenz  Scale (m) | 4.5443  (0.00015128) | 4.833  (7.8623e-05) | 4.272  (4.7932e-04) |
| $\boldsymbol{\chi}^{\boldsymbol{2}}$ | 9319.8 | 19186 | 15912 |

## **Figure S6.** Aerodynamic distribution using next generation impactor (NGI) of LYS-MSP.

3 doses (10 mg) of the same batch LYS-MSP (without washing step) were actuated per replicate using an ICOone® inhaler at 64 L/min. Data are presented as means ± range. The F.P.F was 65.6 ± 4.9%%. The M.M.A.D. was 2.3 ± 0.5 µm, the G.S.D. was 1.68 ± 0.04 and the retention in the inhaler was 4.2 ± 2.1%.
